# Effect of Psychobiotics on Psychometric Tests and Inflammatory Markers in Major Depressive Disorder: Meta-Analysis of Randomized Controlled Trials with Meta-Regression

**DOI:** 10.3390/ph14100952

**Published:** 2021-09-23

**Authors:** Agata Misera, Paweł Liśkiewicz, Igor Łoniewski, Karolina Skonieczna-Żydecka, Jerzy Samochowiec

**Affiliations:** 1 Department of Psychiatry, Pomeranian Medical University, Broniewskiego 26, 71-457 Szczecin, Poland; miseraagata@gmail.com (A.M.); pjliskiewicz@gmail.com (P.L.); samoj@pum.edu.pl (J.S.); 2 Department of Biochemical Sciences, Pomeranian Medical University in Szczecin, Broniewskiego 24, 71-460 Szczecin, Poland; sanprobi@sanprobi.pl; 3 Sanprobi Sp. z o. o. Sp. k. Kurza Stopka 5/C, 70-535 Szczecin, Poland

**Keywords:** depression, probiotics, psychobiotics, microbiota

## Abstract

Probiotics were shown to act positively on gut–brain axis signaling. We aimed to assess the effect of the administration of a new class of probiotics—psychobiotics—using data from individual psychometric scales, markers of the immune system and neuroactive metabolites. Medical databases were searched from database inception until 22 April 2021 for randomized clinical trials in clinically proven Major Depressive Disorder (MDD) patients treated with either probiotics or placebo reporting any psychometric score (PROSPERO registration number: CRD42021253024). Ten studies with 705 randomized participants and 603 analyzed were included. The mean age of individuals was 38.43 ± 12.1 years, predominantly women (*n* = 461, 76.45). The mean study duration was 48.8 ± 12.3 (range = 28–62) days. The dosage ranged between 1 × 10^9^ to 2 × 10^10^ colony forming units (CFU)/day. We found that probiotics might alleviate symptoms of MDD; endpoint data (pooled scores): SMD = −0.292, 95%CI = −0.577 to −0.007, *p* < 0.044; change scores (BDI): SMD = −0.482, 95%CI = −0.854 to –0.109, *p* < 0.011; DM = −4.848, 95%CI = −8.559 to −1.137, *p* < 0.01. The therapy tended to be more effective with time of psychobiotic supplementation (coefficient = −0.12, SE = 0.06, Z = −1.84, *p* = 0.06) and in men (% of females: coefficient = 0.1, SE = 0.06, Z = 1.78, *p* = 0.07). Psychobiotics have great potential in the treatment of MDD. However, no specific strain/strains, dosage or duration of treatment can currently be recommended.

## 1. Introduction

Major Depressive Disorder (MDD) affects approximately 300 million people worldwide and is a common cause of disability and roughly 800,000 suicides per year [1]. Approximately 30% of patients with MDD do not respond to monoaminergic antidepressants [2], suggesting that other biological pathways are involved in MDD etiology. These mechanisms include, i.a., subclinical inflammation [3], hypothalamic–pituitary (HPA) axis dysregulation [4] and altered signaling of neurotrophic growth factors [5]. One very promising hypothesis of MDD pathogenesis is the gut–brain axis (GBA) dysfunction [6], with gut microbiota as a key player. The microbiota was shown to regulate different functions in the central nervous system (CNS), i.a., the promotion of neuropeptides synthesis, regulation of the HPA axis, production of neurotransmitters and tryptophan metabolism [7,8].

Only a few human and animal studies proved the association between gut microbiota and depression [9]. It is hypothesized that the bacterial taxonomic changes observed in patients with MDD are associated with their proinflammatory activity, reduced short-chain fatty acids production, impaired intestinal barrier integrity, skewed neurotransmitter production, impaired carbohydrate and amino acids metabolic pathways [10]. However, in a recently published systematic review (SR), the causation between microbiota and MDD was not confirmed [11]. Consequently, “psychobiotics,” which stands for probiotics that support mental health [12], are currently of great interest and hope for researchers, doctors and patients. Indeed, the use of psychobiotics in patients with MDD has great prospects [11]; however, this procedure requires standardization and thorough mechanistic research. The results of systematic reviews and meta-analyses analyzing the impact of probiotics in patients with depression are not always unambiguous; however, in general, most of them confirm their effectiveness [9,13,14,15,16,17,18,19] but not in every case [20]. It should be emphasized that these meta-analyses pooled the results of studies conducted in populations of healthy people, patients with MDD and persons with various accompanying mental and somatic disorders. Additionally, the sizes of each effect were calculated using data of various psychometric scales, which, from our point of view, might be a methodologically acceptable practice, but, from a clinical point of view, this might not the best to reflect the essence of the assessment of interventions in this group of patients. Moreover, in the meta-analyses carried out so far, the descriptions of mechanisms of action of probiotics are scarce. Therefore, in this meta-analysis, we decided to assess the effect of psychobiotics administration using data from individual psychometric scales, markers of the immune system and neuroactive metabolites to verify the hypothesis that psychobiotics act favorably in MDD and their mechanism of action is related to immunomodulation and metabolic pathways in CNS. In addition, thanks to the meta-regression, we aimed to answer the question of whether it is possible to recommend clinicians a given strain, duration of administration and point variables, which might positively affect this intervention efficacy. We also took into account the papers that appeared after the publication of the latest meta-analysis on the use of probiotics in MDD [17].

## 2. Results

### 2.1. Search Results

**Figure 1:**
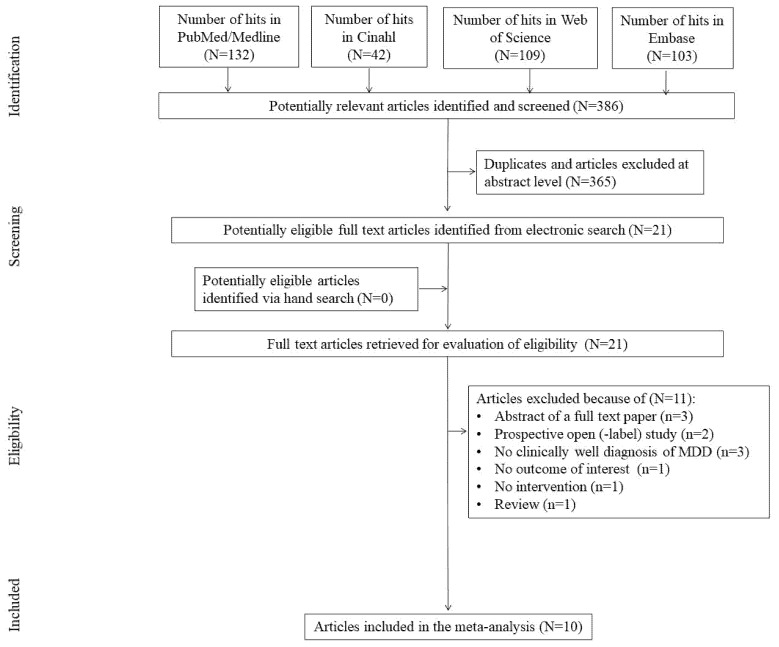
Study flow chart. *N*/*n* = number of studies.

The initial search yielded 386 hits. At first, we excluded 365 studies as for being duplicates and/or after evaluation on the title/abstract level. No additional articles were identified via hand search. Finally, 21 full-text articles were reviewed. Of those, 11 did not fit the inclusion criteria. Primary reasons for exclusion were: abstracts for full-text studies (*N* = 3), no clinically well diagnosis of MDD (*N* = 3) and other than a randomized controlled trial (RTC) design (*N* = 2). We excluded studies being review, with no outcome of interest and no intervention, one per each reason (*N* = 3). Finally, the search yielded 10 studies that were included in the meta-analysis (Figure 1).

### 2.2. Study, Patient and Treatment Characteristics

Studies were predominantly conducted in Iran (*N* = 5) [21,22,23,24,25] but also in Austria (*N* = 2) [26,27], Poland (*N* = 1) [28] and Italy (*N* = 1) [29]. One study was conducted in two clinical centers, in Australia and Netherlands (*N* = 1) [30]. One trial was sponsored by industry (*N* = 1) [29], and no data on sponsorship was available for three studies (*N* = 3) [21,22,25]. In two trials, probiotic administration took place at hospital setting [26,27].

Altogether, 10 studies with 705 participants randomized and 603 analyzed were included [21,22,23,24,25,26,27,28,29,30]. All of patients were diagnosed with MDD, but different clinically well criteria were used, predominantly based on The Diagnostic and Statistical Manual of Mental Disorders (DSM) approaches. In two studies, patients were treatment naïve [29,30], and a subgroup of persons (*n* = 21) in a study by Rudzki [28] started selective serotonin reuptake inhibitors (SSRIs) treatment along with probiotics administration. The mean age of included individuals was 38.43 ± 12.1 years, and predominantly women were included (*n* = 461, 76.45%). The body mass index (BMI) was, on average, in a normal range, i.e., 23.86 ± 7.73 kg/m^2^. The mean study duration was 48.8 ± 12.3 (range = 28–62) days. Different probiotic strains were administered; however, in three studies conducted on the same population [21,22,24], the same Formula (named “Formula 2” for meta-regression purposes) was used: *L. helveticus* R0052 (Collection Nationale de Cultures de Microorganisms; CNCM strain I-1722) and *B. longum* R0175 (CNCM strain I-3470). In two studies (also the same population) [26,27], a mixture (reference Formula, R) of *Bifidobacterium bifidum* W23, *Bifidobacterium lactis* W51, *Bifidobacterium lactis* W52, *L. acidophilus* W22, *Lactobacillus casei* W56, *L. paracasei* W20, *L. plantarum* W62, *Lactobacillus salivarius* W24 and *Lactococcus lactis* W19 was administered. A similar probiotic cocktail was used in a study by Chahwan et al. [30]; however, the studied product also contained *L. acidophilus* W37, *Lactobacillus brevis* W63 and *Lactococcus lactis* W58 instead of *L. acidophilus* W22, *L. paracasei* W20 and *L. plantarum* W62 (for meta-regression purposes also treated as “R” Formula). In two studies [23,25], only the names of the species were shown. The dosage varied between studies and ranged from 1 × 10^9^ to 2 × 10^10^ colony forming units (CFU)/day. In one study [25], the dose was not given (Table 1). Adverse events were reported in participants in both probiotic groups and in placebo-given persons. Details are given in Table 2.

### 2.3. Risk of Bias (ROB)

The mean number of low risk-of-bias assessments in all studies included in the meta-analysis was 6.8 (median = 7) [21,22,23,24,25,26,27,28,29,30]. There were eight studies with the highest number, i.e., seven low ROB assessments [21,22,24,25,26,27,28,30]. Two studies received a score of 6 due to unclear risk of bias in a domain “Incomplete outcome data addressed” [23] or unclear detection bias [29]. 

### 2.4. Effects on Depression Symptomatology

#### 2.4.1. Endpoint Data

**Figure 2:**
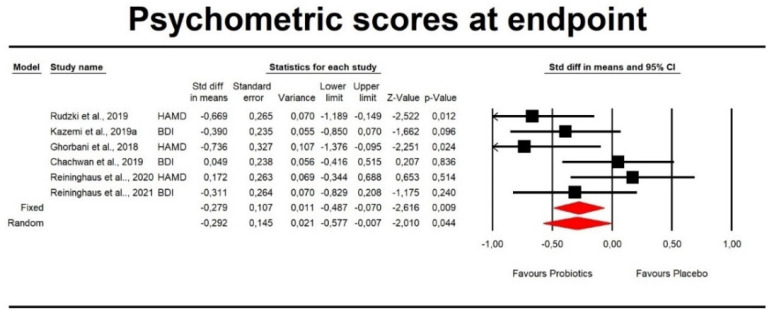
An effect size (random model), SDM, for depression symptoms in clinical scores in persons taking probiotics vs. placebos (controls). Q = 9.197, df(Q) = 5, *p* = 0.101, I^2^ = 45.632.

Using random-effects weights, the standardized difference in means (SDM) for symptomatology of depression evaluated by pooled Beck Depression Inventory (BDI) and Hamilton Depression Rating Scale (HAMD) scores at the endpoint was −0.292 with a 95% confidence interval of −0.577 to −0.007 (*z* = −2.01, *p* < 0.044; Figure 2).

**Figure 3:**
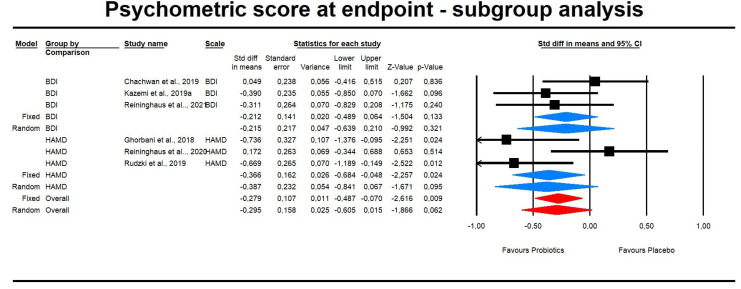
An effect size (random model), SDM, for depression symptoms in clinical scores in persons taking probiotics vs. placebos (controls) subgroup analysis BDI: Q = 1.924, df(Q) = 2, *p* = 0.382, I-squared = 0.0; HAMD: Q = 6.761, df(Q) = 2, *p* = 0.034, I^2^ = 70.41.

**Figure 4:**
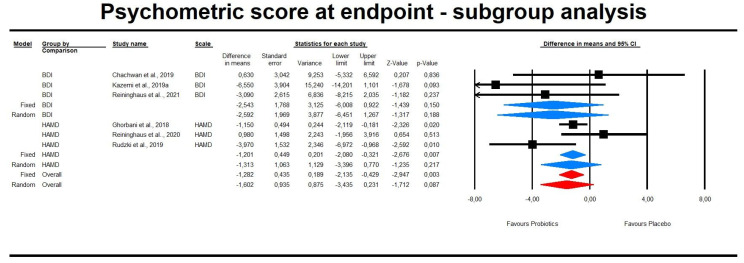
An effect size (random model), DM, for depression symptoms in clinical scores in persons taking probiotics vs. placebos (controls) subgroup analysis BDI: Q = 2.185, df(Q) = 2, *p* = 0.335, I-squared = 8.48; HAMD: Q = 5.399, df(Q) = 2, *p* = 0.067, I^2^ = 62.955.

However, subgroup analyses regarding the type of psychometric scale used demonstrated insignificant results (Figure 3). In the case of difference in means (DM), the effect size of probiotic intervention was also insignificant in the case of subgroups analysis (Figure 4).

**Figure 5:**
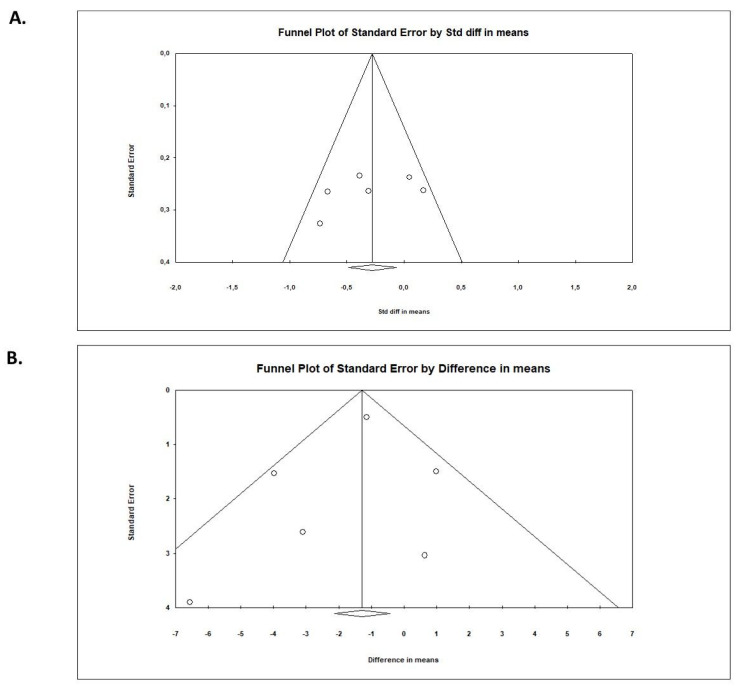
Funnel plots for (**A**) SDM and (**B**) DM for the psychometric score at endpoint.

An Egger’s test did not suggest a publication bias regarding the net effect of probiotics on symptoms of depression (Egger’s test:—SMD: *p* = 0.32;—DM: *p* = 0.55; (Figure 5).

**Figure 6:**
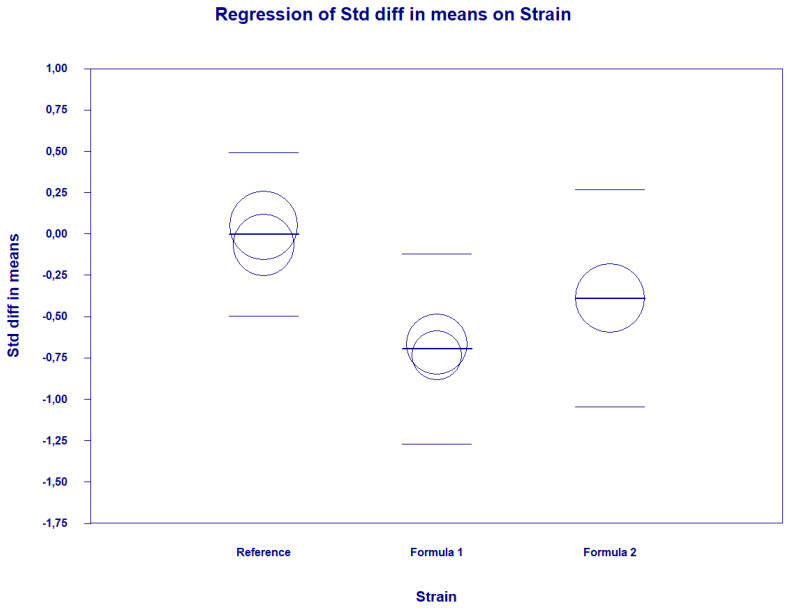
Regression for SDM on strains used in trials. References [26,30], Formula 1 [23,28], Formula 2 [21].

**Figure 7:**
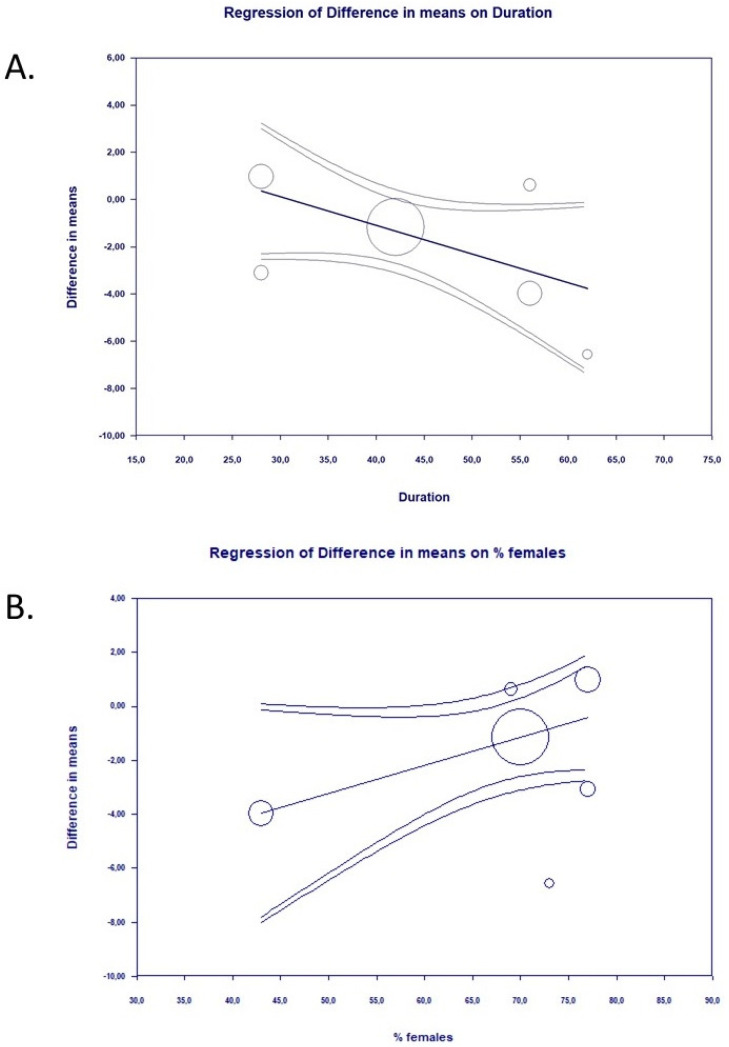
Regression for DM on (**A**) duration of supplementation abs (**B**)% of females participating in the trial.

There were some covariates associated with study-level effects of probiotics on depression symptoms for SDM and DM effect sizes and psychometric score at the endpoint. The significant association between effect size (SDM) and probiotic strains (Formula R as the reference group) was found: probiotic strain “Formula 1” coefficient = −0.69, standard error (SE) = 0.27, Z = −2.55, *p* = 0.01; Probiotic “Formula 2” coefficient = −0.38, SE = 0.29, Z = −1.32, *p* = 0.18. This covariate explained 100% of the variance in the effect size (Figure 6). Other covariates were not linked to the estimated effect size. Duration of intervention: (a) coefficient = −0.0072, SE = 0.01, Z = −0.65, *p* = 0.52; (b) % of females: coefficient = 0.01, SE = 0.01, Z = 1.32, *p* = 0.19; (c) mean age: coefficient = −0.055, SE = 0.14, Z = −0.4, *p* = 0.69 and (d) mean BMI coefficient = 0.03, SE = 0.06, Z = 0.54, *p* = 0.58 The estimates in case of DM were as follows: (a) Probiotic strain “R”; coefficient = −6.42, SE = 4,36, Z =−1.47, *p* = 0.14; Probiotic Strain: Other: coefficient = −1.94, SE = 1.86, Z = −1.04, p = −0.29 (b) duration of intervention: coefficient = −0.12, SE = 0.06, Z = −1.84, *p* = 0.06 (Figure 7A); (c) % of females: coefficient = 0.1, SE = 0.06, Z = 1.78, *p* = 0.07 (Figure 7B) (d) mean age: coefficient = −0.70, SE = 0.45, Z = −1.56, *p* = 0.12 and (e) mean BMI coefficient = 0.88, SE = 0.54, Z = 1.66, *p* = 0.1.

#### 2.4.2. Change Scores

**Figure 8:**
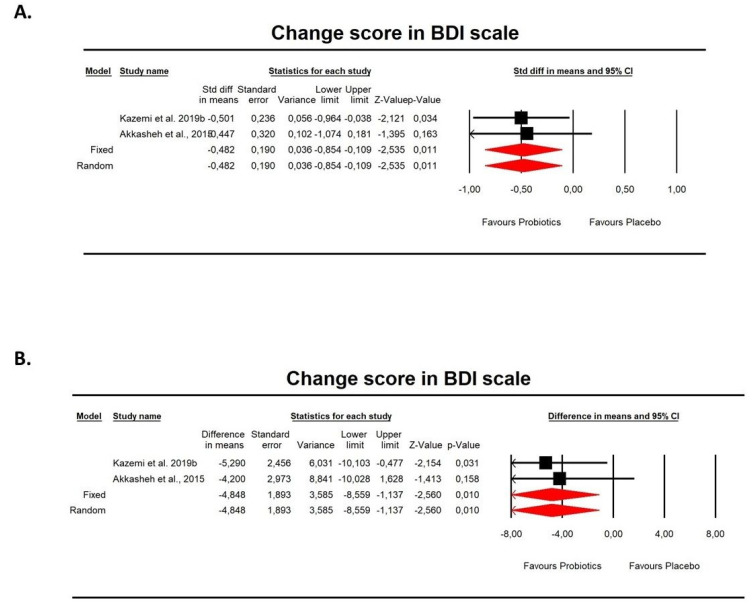
(**A**). An effect size (random model), SDM, for depression symptoms in BDI change score in persons taking probiotics vs. placebos (controls). Overall: Q = 0.019, df(Q) = 1, p = 0.891, I-squared = 0.00; (**B**). An effect size (random model), DM for depression symptoms in BDI change scores in persons taking probiotics vs. placebos (controls). Overall: Q = 0.08, df(Q) = 1, *p* = 0.77, I^2^ = 0.00.

Using random-effects analysis and change score data on the BDI score, we found that probiotics alleviated MDD symptoms using either SDM (−0.482, 95%CI: −0.854 to −0.109, z = −2.535, *p* = 0.011) and DM (−4.848, 95%CI: −8.559 to −1.137, z = −2.56, *p* = 0.001) effect sizes (Figure 8). There were not enough studies to conduct metaregression analyses with the given covariates. However, when we added a study by Saccarello et al. [29], who measured depression symptoms by ZDSD scores, the results turned out to be insignificant in case of SDM: −1.498, 95%CI: −3.348 to 0.369, z = −1.571 (Q = 60.933, df = 2, *p* = 0.00, I^2^ = 96.72).

### 2.5. Effects on Inflammatory Status

Data for inflammatory parameters, cortisol, interleukin 1β (IL-1β), interleukin 6 (IL6), kynurenine and tumor necrosisα factor (TNFα) in particular, were present in two trails [22,28] each.: The effect of probiotics on these parameters were not statistically significant (*p* > 0.05). The SDM were as follows: (a) cortisol: −0.035, 95%CI: −0.409 to 0.340, z= −0.181, *p* = 0.857 (b) IL-1β: 0.167, 95%CI: −0.315 to 0.65, z = 0.68, *p* = 0.497; (c) IL6,:0.199, 95%CI: −0.179 to −0.577, z = 1.032, *p* = 0.302; (d) kynurenine: −0.407, 95%CI: −0.940 to 0.127, z = −1.493, *p* = 0.135 (e) TNFα −0.096, 95%CI: −0.288 to 0.481, z = 0.492, *p* = 0.623. The forest plots are included in Supplementary Figures S1–S5.

## 3. Discussion

The present review included ten randomized clinical trials [21,22,23,24,25,26,27,28,29,30] that evaluated the effectiveness of probiotics in MDD treatment measured by psychometric scales. In five of them, the mechanism of probiotic action was also examined. The antidepressant efficacy of probiotics in MDD was demonstrated in three studies [21,23,25] and the improvement of cognition in two papers [28,30]. Saccarello et al. [29] reported improvements in symptoms of depression, anxiety and cognition along with somatic components, whilst Reininghaus et al. [26] did not report on any beneficial impact of probiotics in the course of MDD. The meta-analysis showed that particular combinations of probiotics or specific species and strains appear to be beneficial in MDD in terms of their effect on the BDI and HAMD pooled psychometric scales, but it is not possible to draw definitive and conclusive conclusions about their effectiveness. The beneficial effect of probiotics is evident in the analysis using change scores in the subjective BDI scale, the magnitude of which is at the middle level. Additionally, the effect size, DM = 4.85, may be of small clinical importance. The results on the BDI and HAMD psychometric scales measured at the endpoint do not confirm the high effectiveness of probiotics due to the small effect size, small statistical significance and weak clinical effect. In addition, serious heterogeneity was observed between the studies in which the HAMD scale was assessed. From among the meta-analyzes published so far, only two works [16,17] analyzed the effectiveness of probiotics in patients with MDD without comorbidities. A recently published updated meta-analysis [17] found a beneficial effect of probiotics in patients with MDD receiving antidepressants but not with probiotic monotherapy. In our study, it was not possible to distinguish such a subgroup (as we subgrouped studied by the psychometric scales), but it should be emphasized that only one study included in the meta-analysis described the population of treatment naïve patients [30]. Moreover, the inference is made more difficult by the fact that half of the analyzed studies took place in Iran, and five of them were carried out on two patient cohorts, which does not ensure adequate representativeness of the analyzed studies.

In this meta-analysis, by means of meta-regression, we failed to demonstrate the efficacy of a particular strain or a combination of different strains. It should be emphasized, however, that probiotic cocktails containing the strains that were used in the works of Chahwan et al. [30], Reininghaus et al. [26] and Reiter et al. [27] differed from each other and their pooling in the metaregression may be a source of error, which, however, does not affect the fact that there is no evidence so far that any particular probiotic strain or combinations of such strains can be recommended in patients with MDD based on the results of the meta-analysis instead of individual RCTs.

The results of the meta-regression suggest that the duration of use might be associated with their greater effectiveness in patients with MDD. Such observation could be explained by the time necessary for changes in the intestinal microbiota along with the administration of probiotics. However, it is difficult to prove such a thesis because only two studies analyzed gut microbiota [26,30], with one study reporting microbiota changes [26]. There is, however, no current contention on whether probiotic treatments could/should successfully alter microbiota composition [31,32]. Nevertheless, probiotics were reported to influence bacterial gene expression and cause anti-inflammatory effects regardless of the influence on microbiota composition [33]. This could explain the cognitive function improvement after probiotic administration. Alternatively, multi-strain probiotics (Ecologic Barrier, Winclove Probiotics, Amsterdam, The Netherlands) can improve gut barrier function in vitro [34] and in humans [35], which can be also related to a decrease in systemic inflammation, which can improve symptoms of MDD.

We observed that the effectiveness of probiotics has a tendency to be inversely proportional to the percentage of women participating in the study. Epidemiological studies have shown that depressive disorders occur approximately two or three times more frequently in women than men [36,37]. Moreover, the clinical characteristics and treatment outcomes of depressive disorders in women are different from those in men [37,38]. Interestingly, the composition of the microbiota in men and women suffering from MDD [39] differ from one another, which might stand for different probiotic efficacy.

In mechanistic studies, Rudzki et al. and Kazemi et al., 2019a [21,28] observed a decreased blood kynurenine concentration. Some kynurenine catabolites may have a role in patients with MDD due to its neurotoxic and neurodegenerative effects [40]. Metagenomic analysis showed a disorder of tryptophan synthesis in patients with MDD [41,42,43,44]. It seems, therefore, that probiotic administration may have influenced tryptophan metabolism. Other analysis [22] found a clinically significant decrease in urine cortisol concentration, Akasheh et al. [25] and Reiter et al. [27] observed anti-inflammatory effects of probiotics which, however, have not been confirmed in other studies [28,45]. Heidarzadeh-Rad et al. [24] reported an increased brain-derived neurotrophic factor (BDNF) level, which was shown to correlate with antidepressant response in MDD.

Based on experimental studies and results mentioned above, it can be concluded that probiotics have the potential to influence various mechanisms of etiopathology of depression involving inflammation, neurotransmitters and the hypothalamic–pituitary–adrenal (HPA) axis. However, the meta-analysis did not confirm the effectiveness of the use of probiotics in regulating the parameters associated with the HPA axis (cortisol level), inflammation (interleukins, TNF) and tryptophan degradation pathway (kynurenine). In another meta-analysis, Amirani et al. [19] reported that taking probiotics by patients with different psychiatric disorders (not only MDD) had beneficial effects on C-reactive protein (CRP), IL-10 and malondialdehyde (MDA) levels, but it did not affect other markers of inflammation (TNF-alpha, IL-1B) and oxidative stress. However, due to the ambiguous results of the research and the high heterogeneity of the studied populations, it can be said that the clinical mechanism of the action of probiotics in improving the symptoms of MDD remains the subject of speculation. Finally, it should be stated that taking probiotics is well tolerated, safe and poses no risk in patients with MDD. Additionally, the intervention is not associated with significant side effects.

Our systematic review and meta-analysis has many strengths. We applied a rigorous and repeatable methodology. Our search strategy was described in detail; moreover, unlike other studies, we qualified the data obtained in RCT in patients with medically confirmed MDD, and we did not take into account patients with comorbidities and healthy patients who were assessed for depressive symptoms. Finally, we conducted subgroup analyses to assess the treatment effect depending on the psychometric scale used, and we also conducted a risk of bias analysis, reasons for study discontinuations and a very detailed analysis of adverse effects. We also performed the Egger’s test and meta-regression analysis.

It should also be emphasized that our systematic review and meta-analysis also have significant limitations. The number of studies and the size of the study groups are both small. The methodological heterogeneity is significant; the five analyzed studies concern two small cohorts; finally, Iranian patients are over-represented in these studies. Mechanistic research is quite sparse. Researchers have rarely analyzed the gut microbiota, and the immunome or metabolome has not been analyzed at all.

## 4. Materials and Methods

### 4.1. Search Strategy and Inclusion Criteria

There were two independent authors (KSZ and PL) who searched PubMed/Embase/Cinahl and Web of Science from database inception until 22/04/2021 without language restriction for RCTs that compared adjunctive probiotics with placebo to counteract depression symptomatology.

The following search strings were used:

Embase—(‘depression’/exp OR ‘central depression’ OR ‘clinical depression’ OR ‘depression’ OR ‘depressive disease’ OR ‘depressive disorder’ OR ‘depressive episode’ OR ‘depressive illness’ OR ‘depressive personality disorder’ OR ‘depressive state’ OR ‘depressive symptom’ OR ‘depressive syndrome’ OR ‘mental depression’ OR ‘parental depression’ OR ‘major depression’/exp OR ‘depression, major’ OR ‘depression, unipolar’ OR ‘depressive disorder, major’ OR ‘major depression’ OR ‘major depressive disorder’ OR ‘major depressive episode’ OR ‘unipolar depression’ OR ‘unipolar disorder’) AND (‘probiotic agent’/exp OR ‘probiotic’ OR ‘probiotic agent’ OR ‘probiotics’ OR ‘synbiotic agent’/exp OR ‘synbiotic’ OR ‘synbiotic agent’ OR ‘synbiotics’ OR ‘bifidobacterium’/exp OR ‘bifidobacterium’ OR ‘lactobacillus’/exp OR ‘lactobacillus’ OR ‘lactobacteria’ OR ‘lactobacilli’) AND (‘placebo’/exp OR ‘placebo’ OR ‘placebos’) AND ‘randomized controlled trial’/exp.

PubMed—(MDD OR depression) AND (probiotic* OR probio* OR psychobiotic* OR bifido* OR lacto* OR synbiotic*) AND (RCT OR random* OR placebo*).

Cinahl/Web of Science—(MDD OR depression) AND (probiotic OR psychobiotic OR bifidobacterium OR lactobacillus OR synbiotic) AND (RCT OR random OR placebo).

Finally, we additionally did the manual review of reference lists from eligible reviews to supplement properly the electronic search.

Inclusion criteria were:

1. clinical diagnosis of MDD,
2. probiotic or psychobiotic or symbiotic administration,
3. RCT design,
4. meta-analyzable endpoint/change score data on any of the psychometric tests.

We excluded studies in which the study participants had any concomitant disease, were practicing heavy exercise and/or maintained a strict diet. We also did not include studies in pregnancy and postpartum periods.

The study was registered in the Prospero database under the number CRD42021253024.

### 4.2. Data Abstraction

Data on study design, risk of bias [46], patient, illness and treatment characteristics from each study were independently extracted in accordance with the Preferred Reporting Items for Systematic Reviews and Meta-Analyses (PRISMA) standard [47] by two independent investigators (KSZ and PL). Whenever data were missing for the review, authors were contacted for additional information. Inconsistencies were resolved by consensus, and the principal investigator was involved (AM).

### 4.3. Outcomes

A primary outcome was data on the symptoms of depression expressed by means of any of the clinically well tools. Secondary outcomes included any of the inflammation-related parameters: cortisol, IL-6, IL-1β, TNF-α, and the neuroactive metabolite—kynurenine.

### 4.4. Data Synthesis and Statistical Analysis

We conducted a random-effects [48] MA of outcomes for which ≥2 studies contributed data, using Comprehensive Meta-Analysis V3 (http://www.meta-analysis.com). Outcomes from the same study group were meta-analyzed only once. In the case of at least 3 studies included, we inspected funnel plots and used Egger’s regression test [49] and, where appropriate, the Duval and Tweedie’s trim and fill method [50] to quantify whether publication bias could have influenced the results. We explored study heterogeneity using the chi-square test of homogeneity, with *p* < 0.05 indicating significant heterogeneity. All analyses were two-tailed with alpha = 0.05.

Group differences in continuous outcomes were analyzed as the pooled standardized difference in means (SDM)/difference in means (DM) in either endpoint scores (preferred) or change from endpoint to baseline using observed cases (OC). Categorical outcomes were analyzed by calculating the pooled risk ratio (RR), using intention to treat (ITT) data preferably.

We conducted subgroup and exploratory maximum likelihood random-effects meta-regression analyses of the co-primary and secondary outcomes. Meta-regression variables included: (i) age (mean), (ii) BMI (mean), (iii) supplementation time, (iv) gender and (v) probiotic strains used.

## 5. Conclusions

Based on the results of the analyzed studies, it can be said that probiotics have great potential in the treatment of MDD, especially as an adjunct to traditional therapy. However, no specific probiotic strain or their combinations, dosage or duration of treatment can currently be recommended. The mechanism of action of probiotics in MDD has also not been defined. It is necessary to conduct well-planned clinical trials that take the appropriate number of patients into account and to study the composition of the microbiota, metabolome, immunome and other markers related to MDD.

## Figures and Tables

**Table 1:** Study characteristics.

Study Description				Intervention						Sample Characteristics		
Reference/Country/Sponsorship/Registration No.	Blinding/ Setting	Diagnostic Criteria	Treatment	N total Randomized/ Analyzed	Number of Patients Per Study Group	Probiotic Strain	Duration (Days)	Probiotic Daily Dose (CFU)	Comparator	Age (Mean ± SD)	Females (*n*, %)	BMI (Mean ± SD)
Rudzki et al., 2019 [28]/Poland/Academia/ ClinicalTrials.gov: NCT02469545 (access date 20 August 2021)	DB/ Outpatient	DSM IV-R	Current SSRI monotherapy (*n* = 9), beginning of SSRIs treatment (*n* = 21)	79/60	S: *n* = 30, C: *n* = 30	*L. plantarum* 299v	56	2 × 10^10^	Placebo	Whole group: 39.02 ± 11.0; S: 39.13 ± 9.96; C: 38.9 ± 12;	S: *n* = 23 (76.7); C: *n* = 20 (66.7)	Whole group: 23.82 ± 3.47; S: 24.09 ± 3.76; C: 23.55 ± 3.13
^$^ Kazemi et al., 2019a,b [21,22]; /Iran/nd/ www.irct.ir: IRCT2015092924271N1 (access date 20 August 2021)	DB/ Outpatient	ND	Sertraline, fluoxetine, citalopram or amitriptyline ≥3 months before the trial.	74/74 for BDI; 54 other outcomes	S: *n* = 38 for BDI; 28 other outcomes, C: *n* = 36 for BDI; 26 other outcomes	*L. helveticus* R0052 (CNCM strain I-1722) and *B. longum* R0175 (CNCM strain I-3470)	2 months	1 × 10^10^	Placebo	Whole group (ITT): 36.07 ± 5.84; S:36.15 ± 7.85; C: 36 ± 8.47	S (ITT): *n* = 27 (71); C (ITT): *N* = 24 (66.6)	Whole group (ITT): 26.35 ± 4.57; S (ITT): 26.11 ± 4; C (ITT): 26.61 ± 4.97
^$^ Heidarzadeh-Rad et al., 2020 [24] /Iran/academia/ www.irct.ir: IRCT2015092924271N1 (access date 20 August 2021)	DB/ Outpatient	ND	Sertraline, fluoxetine, citalopram or amitriptyline ≥3 months before the trial.	74/53 ^	S: *n* = 28, C: *n* = 25	*L. helveticus* R0052 (CNCM strain I-1722) and *B. longum* R0175 (CNCM strain I-3470)	2 months	1 × 10^10^	Placebo	Whole group: 36.95 ± 8.24; S: 37.8 ± 7.9/ C: 36.0 ± 8.5	S: *n* = 20 (71.4); C: *n* = 15 (60)	Whole group: 26.69 ± 4.35; S: 26.6 ± 4.2; C: 26.8 ± 4.5
Ghorbani et al., 2018 [23] /Iran/academia/ Shahid Behesti University of Medical Science ethics committee (code: 1394/s/35029)	DB/ Outpatient	DSM-V	Fluoxetine (20 mg/d) for 4 weeks before study	40/40	S: *n* = 20, C: *n* = 20	*L. casaei*, *L. acidofilus*, *L. bulgarigus*, *L. rhamnosus*, *B. breve*, *B. longum*, *S. thermophilu* and fructooligosaccharide	42	*L. casaei* 3 × 10^8^; *L. acidofilus* 2 × 10^8^, *L. bulgarigus* 2 × 10^9^, *L. rhamnosus* 3 × 10^8^, *B. breve* 2 × 10^8^, *B. longum* 1 × 10^9^, *S. thermophilus* 3 × 10^8^ and 200 mg fructooligosaccharide	Placebo	Whole group: 34.97 ± 4.68; S: 34.45 ± 3.95; C: 35.5 ± 5.27	S: *n* = 14 (70); C: *n* = 14 (70)	Whole group: 24.07 ± 5.03; S: 24.04 ± 5.25; C: 24.11 ± 4.81
Chahwan et al., 2019 [30]/ Australia and Netherlands/academia/ ANZCTR, Trial ID: ACTRN12615001081505	TB/ Outpatient	BDI-II	None	71/71	S: *n* = 34, C: *n* = 37; Non-depressed control: *n* = 20 (microbiome analysis)	*B. bifidum* W23, *B. lactis* W51 and W52, *L. acidophilus* W37, *L. brevis* W63, *L.casei* W56, *L. salivarius* W24, *L. lactis* W19 W58	56	1 × 10^10^	Placebo	Whole group (S + C): 36.04 ± 12.07; S: 36.65 ± 11.75/ C: 35.49 ± 12.34	S: *n* = 21 (61.76); C: *n* = 28 (75.67);	ND
Akkasheh et al., 2015 [25]/Iran/nd/ www.irct.ir: IRCT2014060717993N1 (access date 20 August 2021)	DB/ Outpatient	DSM-IV; ≥15 in HAM-D 17-item	ND	40/40	S: *n* = 20, C: *n* = 20	*L. acidophilus*, *L. casei* and *B. bifidum*	56	1 capsule daily, content of each—bacteria 2 × 10^9^ CFU/g, weight of capsules was not given.	Placebo	Whole group: 37.25 ± 10.38; S: 38.3 ± 12.1/ C: 36.2 ± 8.2	ND	Whole group: 26.95 ± 5.18; S: 27.6 ± 6.0; C: 26.3 ± 4.1
Saccarello et al., 2020 [29]/Italy/industry/ ClinicalTrials.gov: NCT03932474 (access date 20 August 2021)	DB/ Outpatient	ICD-10 (F33.0); ZSDS score 41–55	None	90/87	S: *n* = 43, C: *n* = 44	Lactobacillus plantarum Heal 9 + SAMe	42	1 × 10^9^ + 200 mg SAMe	Placebo	Whole group: 48.1 ± 11.25; S: 48.6 ± 10.67/C: 47.5 ± 11.9	S: *n* = 38 (84.4); C: *n* = 35 (79.5)	Whole group: 24.3 ± 5.44; S: 24.1 ± 6.16; C: 24.5 ± 4.64
Reininghaus et al., 2020 * [26]/ Reiter et al., 2020 * [27]/ Austria/academia/ ClinicalTrials.gov: NCT03300440 (access date 20 August 2021)	DB/ Inpatient	M.I.N.I.	125 mg D-Biotin (vitamin B7), 30 mg of common horsetail, 30 mg of fish collagen and 30 mg of keratin plus matrix. Psychiatric treatment: ^##^	82/61	S: *n* = 28, C: *n* = 33	*B. bifidum* W23, *B. lactis* W51, *B. lactis* W52, *L. acidophilus* W22, *L. casei* W56, *L. paracasei* W20, *L. plantarum* W62, *L. salivarius* W24, *L. lactis* W19 and FOS	28	7.5 × 10^9^	Placebo	Whole group: 41.44 ± 12.92; S: 43.00 ± 14.31; C: 40.11 ± 11.45	S: *n* = 20 (71.4); C: *n* = 27 (81.8)	Whole group: 25.99 ± 6.65; S: 26.29 ± 5.78; C: 25.74 ± 7.29

^$^—the same cohort * the same cohort; ^ 1 patient dropped out due to insufficient volume of collected serum; CFU—colony forming unit; SD—standard deviation; BMI—body mass index; *n*—number; DB—double blind; DSM—Diagnostic and Statistical Manual of Mental Disorders; SSRI—selective serotonin reuptake inhibitor; S—study group; C—control group; ND—no data; BDI—Beck Depression Inventory; ITT—Intention To Treat; TB—Triple blind; HAM-D 17-item—Hamilton rating scale for depression; ICD—International Classification of Diseases; ZSDS—Zung Self-Rating Depression Scale; SAMe—S-Adenosylmethionie; M.I.N.I.—Mini-International Neuropsychiatric Interview; NDRI—norepinephrine–dopamine reuptake inhibitor; SNRIs—serotonin–norepinephrine reuptake inhibitors; TZA—tri- and tetracyclic antidepressants, FOS—fructooligosaccharides, ^##^: anticonvulsants, atypical antipsychotics, benzodiazepines and hypnotics, glutamatergic antidepressants, low-potency antipsychotics, melatonin-like antidepressants, mixed preparation of antidepressant and antipsychotic, noradrenergic and specific serotonergic antidepressants, NDRI, SSRIs, SNRIs and TZA.

**Table 2:** Major study outcomes and adverse effects reported.

Reference/Country/Sponsorship	Major Study Focus/Outcome	Discontinuation: All Cause		Discontinuation: Adverse Effects		Adverse Effects (%)	
		PRO	PBO	PRO	PBO	PRO	PRO
Rudzki et al., 2019 [28]/Poland/Academia	Influence of psychobiotic administration on cognitive, affective and immune parameters of Major Depressive Disorder (MDD) patients treated with SSRIs /improvement of cognitive performance and decrease of blood kynurenine concentration after psychobiotic treatment.	10/40	9/39	0	0	Headache (13.3), Loose Stool (3.3), Flatulence (3.3), Palpitations (3.3).	Headache (6.6), Vertigo (3.3), Tremor (3.3), Loose Stool (3.3).
^$^ Kazemi et al., 2019a [21]/Iran/nd	Influence of psychobiotic administration on Beck Depression Inventory (BDI) score; kynurenine, tryptophan and Branch chain amino acids (BCAAs) blood concentration in MDD patients treated with antidepressive agents/improvement in BDI score, decrease in the kynurenine:tryptophan ratio adjusted for serum isoleucine.	10/36	10/38	5	0	Gi complaints (5.56), Nausea (2.78), Fever and body aches (2.78), Increased appetite (13.89).	0
^$^ Kazemi et al., 2019b [22]/Iran/nd	Effect of psychobiotic treatment on blood pro-inflammatory cytokines and the urinary cortisol level in MDD patients treated with antidepressive agents/clinically (not statistically) significant change of urine cortisol concentration.						
^$^ Heidarzadeh-Rad et al., 2020 [24]/Iran/academia	Effect of psychobiotics on serum BDNF in MDD patients treated with antidepressive agents/increase in BDNF, which was inversely correlated with depression severity.	11 ^/36	10/38	5	0	Gi complaints (5.56), Nausea (2.78), Fever and body aches (2.78), increased appetite (13.89).	0
Ghorbani et al., 2018 [23]/Iran/academia	Influence of synbiotic on HAM-D 17-item score in MDD patients treated with fluoxetine/significant decrease of HAM-D 17-item score.	0/20	0/20	0/20	0/20	Bloating (20), Diarrhea (10), Abdominal Cramps (15), Nausea (20).	Bloating (5), Nausea (10).
Chahwan et al., 2019 [30]/Australia and Netherlands/academia	The primary aim: influence of probiotic on the reduction in depressive symptoms. A secondary aims: 1. the treatment response depending on baseline levels of depression; 2. effects of the probiotics on cognitive reactivity, 3. gut microbiota analysis/greater reduction in cognitive reactivity in probiotic group.	11/34	13/37	0/34	0/37	Nausea (32.35), Abdominal/Stomach Pain/Discomfort (26.47), Dehydration (26.47), Drowsiness (20.59) ^#^, Bloating (14.71), Flatulence (11.76), Change in bowels (11.76), Dizzy (8.82), Constipation (8.82), Diarrhoea (5.88), Rash/Itchy (5.88), Vomiting (5.88), Unpleasant Taste (5.88), Headache (5.88).	Nausea (16.22), Abdominal/Stomach Pain/Discomfort (18.92), Dehydration (24.32), Drowsiness (2.7), Bloating (2.7), Flatulence (5.41), Change in bowels (2.70), Dizzy (10.81), Diarrhoea (8.11), Rash/Itchy (5.41), Vomiting (2.7), Unpleasant taste (2.7), Dry Mouth (13.51).
Akkasheh et al., 2015 [25]/Iran/nd	Effects of probiotics on symptoms of depression and metabolic parameters/ favorable effect on BDI and decrease serum insulin, HOMA-IR, serum hs-CRP; increase GSH.	3/20	2/20	ND	ND	ND	
Saccarello et al., 2020 [29]/Italy/industry	Primary: influence of symbiotic on overall symptomatology of depression. Secondary: 1. effects of treatment on symptoms associated with depression, 2. overall health status and 3. safety assessment/significantly improved symptoms of depression, anxiety and cognitive and somatic comoponents.	2/45	0/44	0/45	0/44	Reduced appetite and low mood (4.44)	Rash, rrythema and itching (2.22);
* Reininghaus et al., 2020 [26]/Austria/academia	Influence of treatment on overall symptomatology of depression and gut microbiota/No significant effect of probiotics on clinical symptoms. Observed effect on gut microbiota.	14/42	7/40	0/42	0/40	ND	
* Reiter et al., 2020 [27]/Austria/academia	Influence of probiotics on the expression of inflammation-related genes/Decreased expression of IL-6.						

^$^—the same cohort * the same cohort; ^ 1 patients dropped out due to insufficient volume of collected serum, ^#^—significant in comparison to placebo; PRO—psychobiotic; PBO—Placebo; ND—no data; HAM-D 17-item—Hamilton rating scale for depression; HOMA-IR—homeostasis model assessment of insulin resistance; hs-CRP—high sensitivity C-reactive protein; GSH—total glutathione; IL—interleukin.

## Data Availability

Data is available upon request.

## Supplementary Materials

The following are available online at https://www.mdpi.com/article/10.3390/ph14100952/s1, Figure S1: An effect size, SDM, for cortisol in persons taking probiotics vs. controls. Q = 0.886, df(Q) = 1 *p* = 0.347, I-squared = 0.0, Figure S2: An effect size, SDM, for Il-1β in persons taking probiotics vs. controls. Q = 0.074, df(Q) = 1 *p* = 0.785, I-squared = 0.0, Figure S3: An effect size, SDM, for Il-6 in persons taking probiotics vs. controls. Q = 0.020, df(Q) = 1 *p* = 0.887, I-squared = 0.0, Figure S4: An effect size, SDM, for WC in persons taking probiotics vs. controls. Q = 2.381, df(Q) = 1 *p* = 0.123, I-squared = 58.007, Figure S5: An effect size, SDM for WC in persons taking probiotics vs. controls. Q = 0.074, df(Q) = 1 *p* = 0.785, I-squared = 0.0.
