# Supplementary figures and images for: Effect of Psychobiotics on Psychometric Tests and Inflammatory Markers in Major Depressive Disorder: Meta-Analysis of Randomized Controlled Trials with Meta-Regression
Source: Pharmaceuticals (Basel). 2021 Sep 23;14(10):952. doi: 10.3390/ph14100952 (PMC8541446; doi:10.3390/ph14100952)

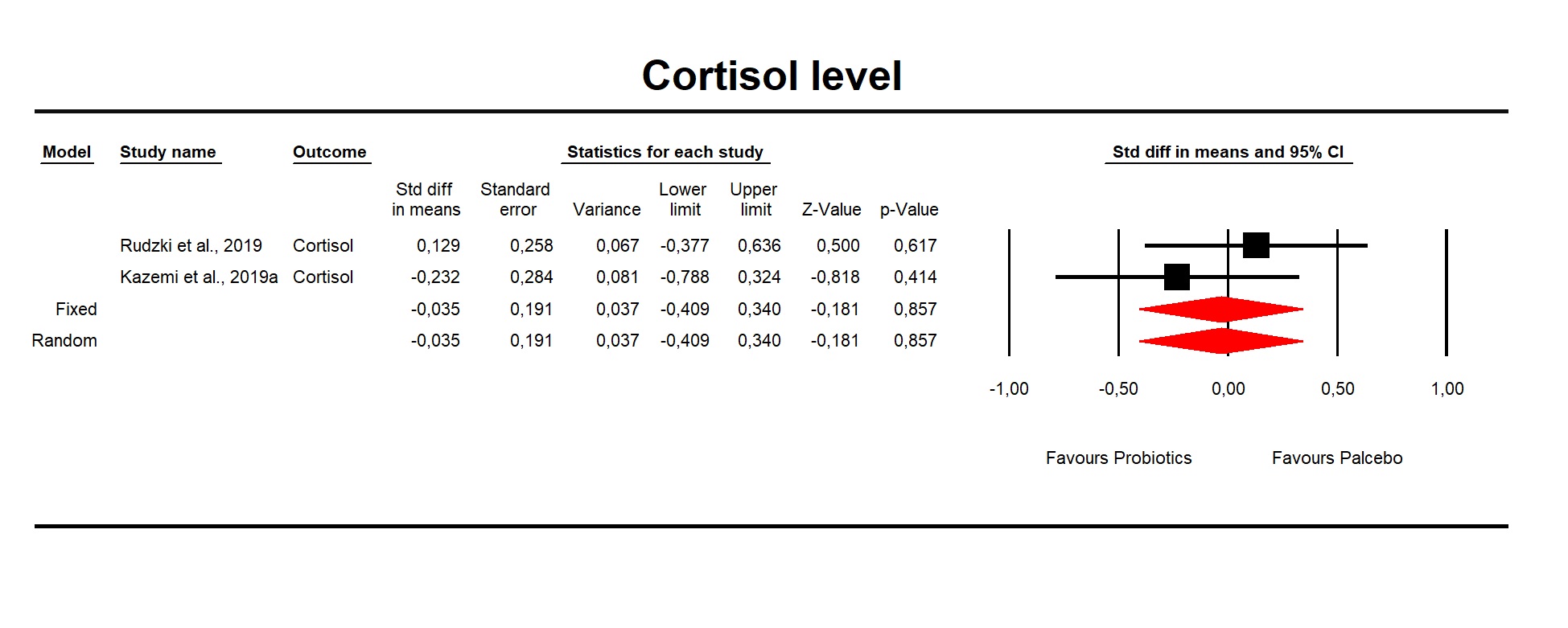

Supplement: Supplementary file 1 [file pharmaceuticals-14-00952-s001.zip › Supplementary Figure S1.jpg]

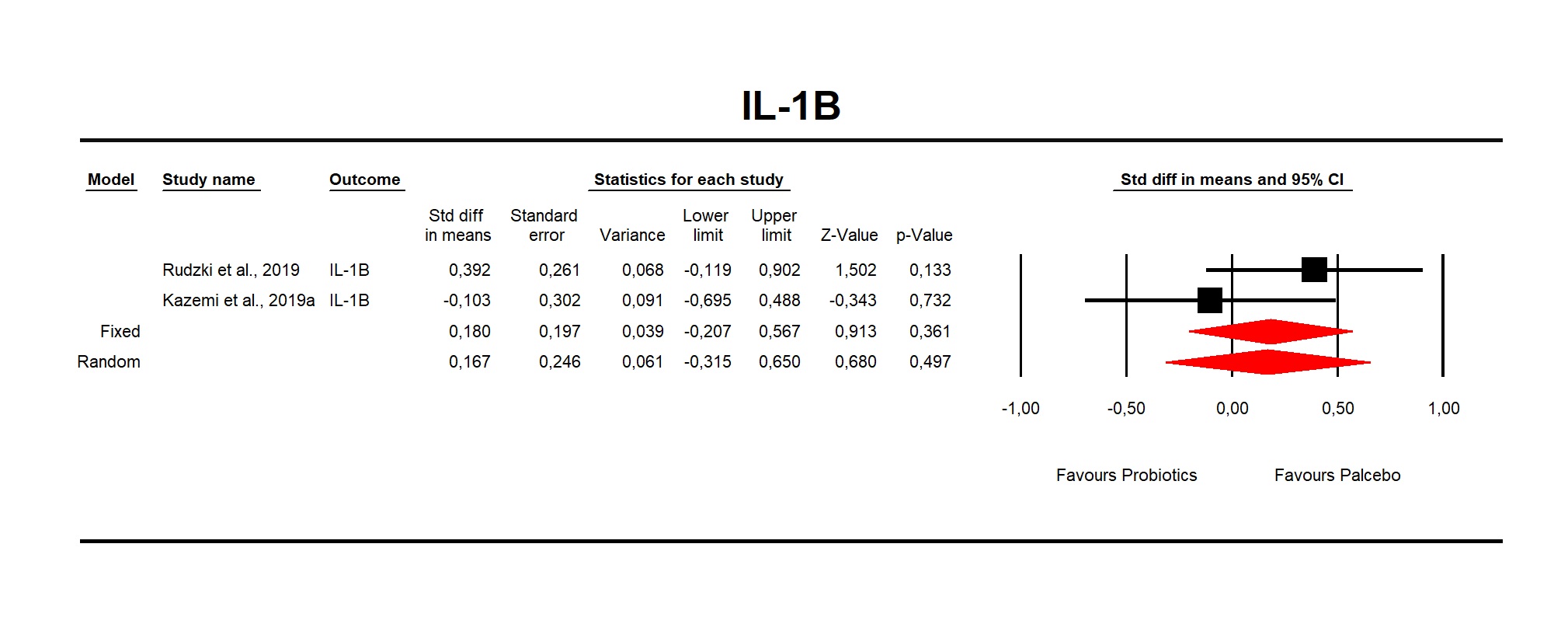

Supplement: Supplementary file 1 [file pharmaceuticals-14-00952-s001.zip › Supplementary Figure S2.jpg]

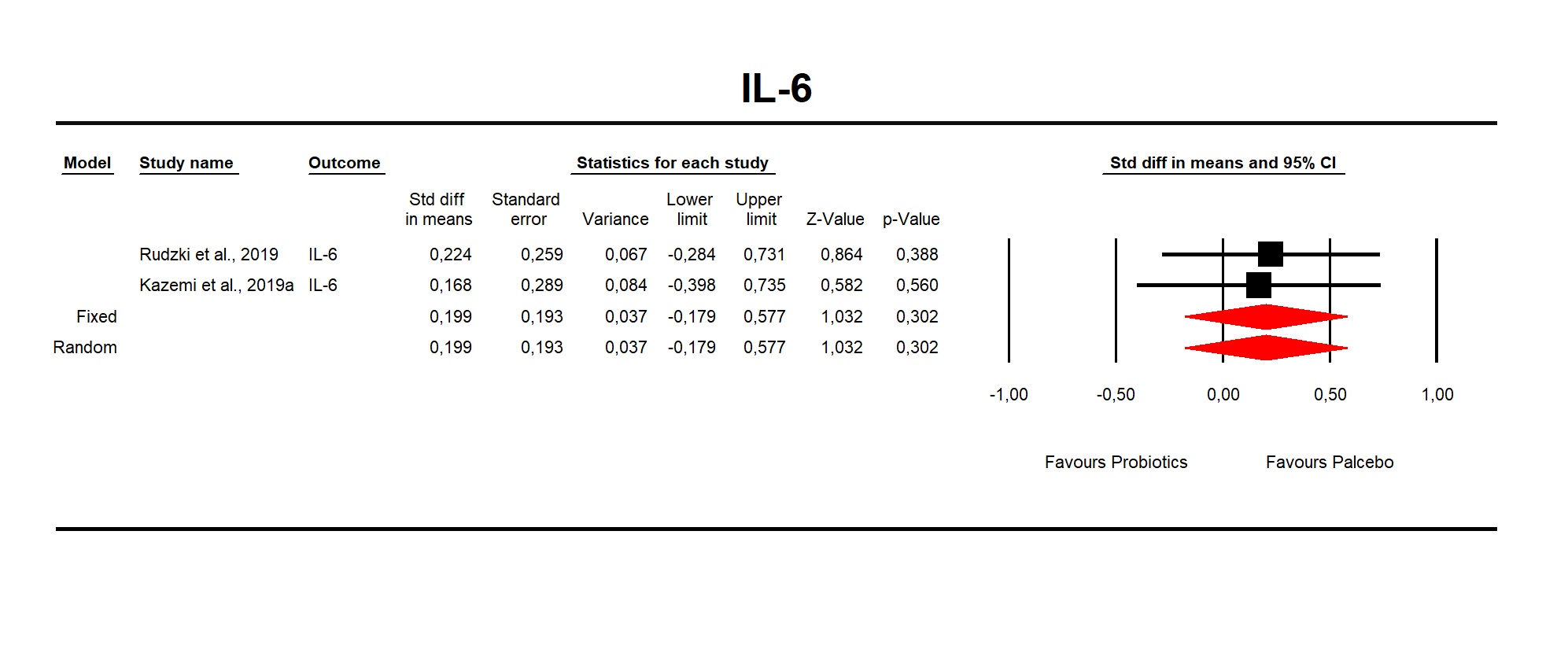

Supplement: Supplementary file 1 [file pharmaceuticals-14-00952-s001.zip › Supplementary Figure S3.jpg]

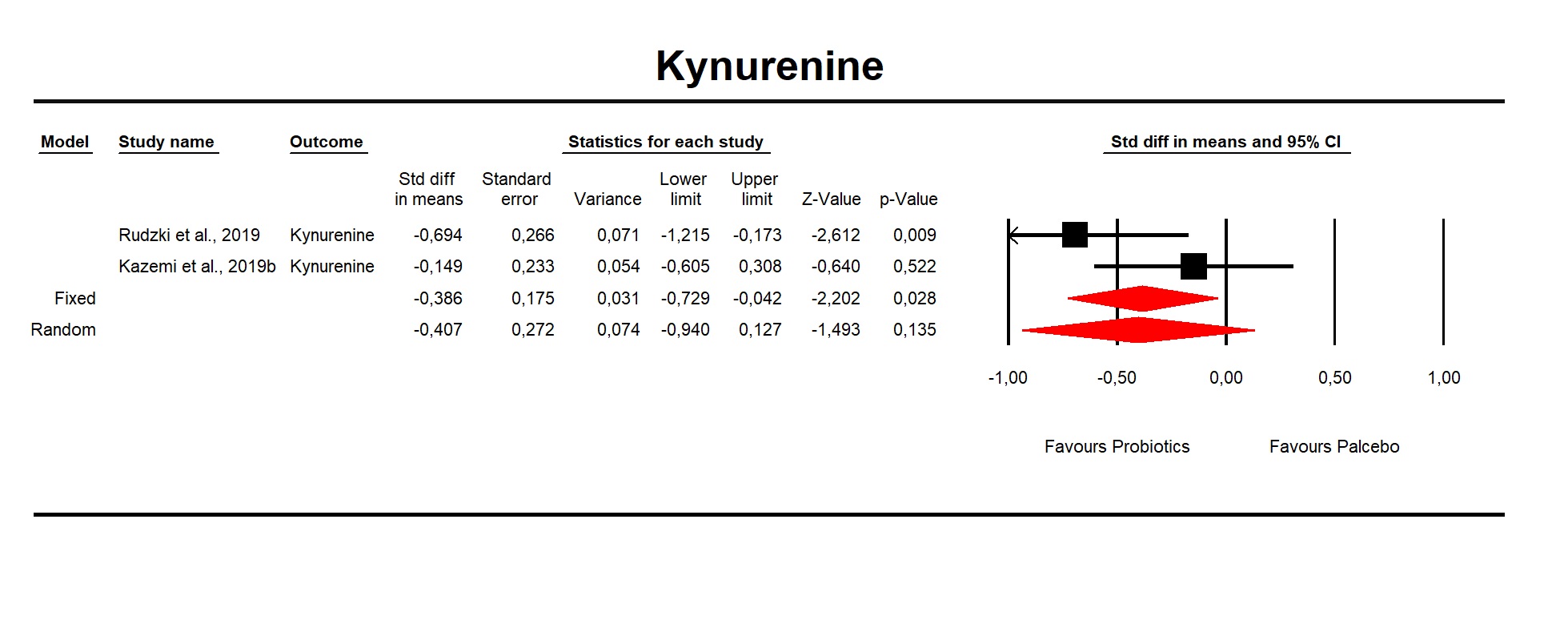

Supplement: Supplementary file 1 [file pharmaceuticals-14-00952-s001.zip › Supplementary Figure S4.jpg]

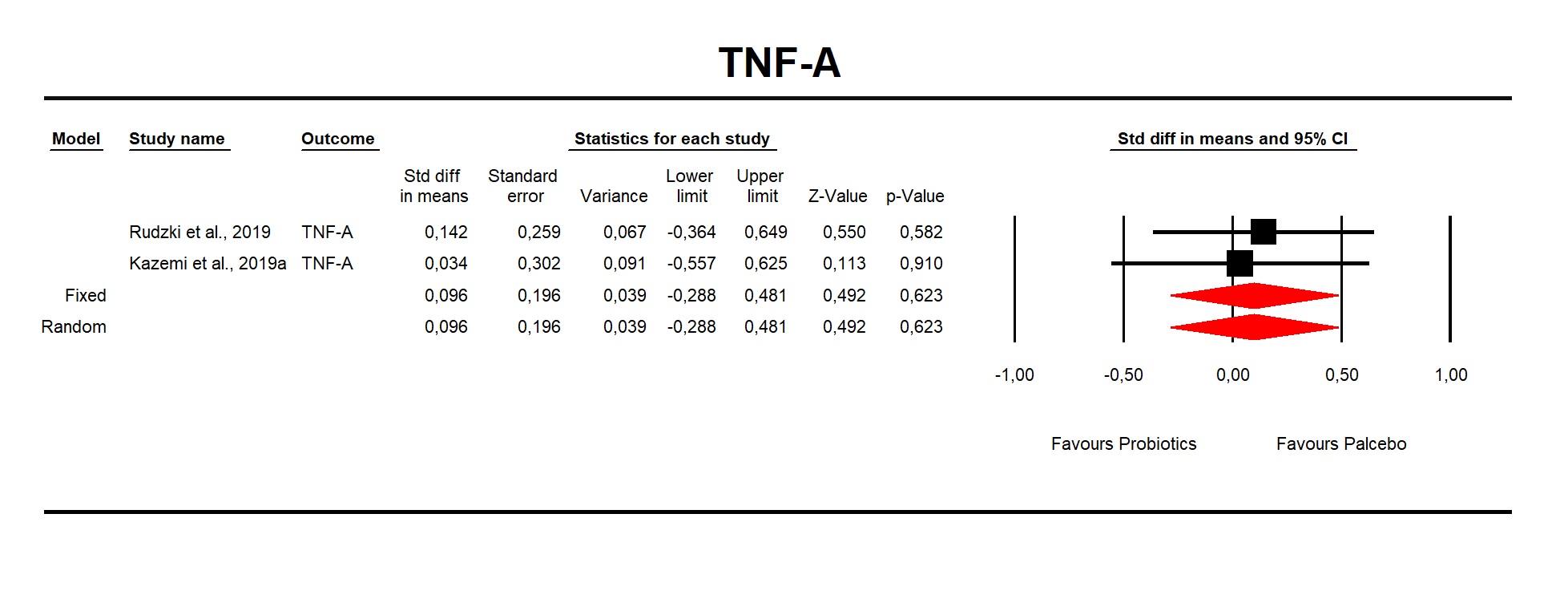

Supplement: Supplementary file 1 [file pharmaceuticals-14-00952-s001.zip › Supplementary Figure S5.jpg]
